# Role of Cardiovascular Computed Tomography in Acute Coronary Syndromes During the COVID-19 Pandemic-Single Center Snapshot Study

**DOI:** 10.3389/fcvm.2021.665735

**Published:** 2021-05-11

**Authors:** Mirvat Alasnag, Waqar Ahmed, Ibrahim Al-Nasser, Khaled Al-Shaibi

**Affiliations:** ^1^Cardiac Center, King Fahd Armed Forces Hospital, Jeddah, Saudi Arabia; ^2^Radiodiagnostics Department, King Fahd Armed Forces Hospital, Jeddah, Saudi Arabia

**Keywords:** cardiac computed tomography, COVID-19, acute coronary syndrome, infection, revascularization

## Abstract

**Background:** In clinical practice, cardiac computed tomography (CCT) has a limited role in acute coronary syndromes (ACS). Several trials evaluated CCT in low and intermediate risk patients presenting to the emergency room (ER) and noted that it was both safe and feasible. During the COVID19 pandemic, it is imperative to adopt a pathway for the evaluation of ACS that permits early discharge, reduces invasive coronary angiography and limits exposure of healthcare workers. Here, we present a single center experience by which CCT was incorporated in the clinical pathway of patients presenting to the ER with chest pain and ACS.

**Methods:** This is a snapshot study of the first 27 patients who underwent CCT immediately after the lockdown in the city of Jeddah. ST elevation myocardial infarctions and hemodynamically unstable patients were excluded. Those with unstable angina or a Non-ST elevation myocardial infarction were screened for COVID19. The patients' COVID19 status and the results of the CCT were then used to determine the treatment strategy. Patient predisposition, hospital stay and exposure of staff are collected and reported.

**Results:** All CCT images were interpretable with no limitations or significant artifact. CCT identified critical disease in 7 patients (26%), normal epicardial coronary arteries in 11 (41%) and mild to moderate disease in 9 (33%). All patients with normal or mild to moderate disease were assigned to a conservative strategy and discharged within 24 h. Those with a NSTEMI and critical anatomy were assigned to an additional invasive evaluation with subsequent revascularization. During the course of this study, no transmission to healthcare workers occurred.

**Conclusion:** CCT enabled 80% of patients to be discharged within the first 24 h, the majority of whom were discharged from the emergency room. It was able to identify critical anatomy facilitating appropriate revascularization. This snapshot study warrants exploration of the role of CCT in ACS further particularly since the latest European Society of Cardiology's Non-STEACS guidelines suggest a role for CCT in the evaluation of low risk ACS.

## Introduction

The SARS-CoV-2 [Corona Virus Disease 2019 (COVID19)] pandemic has posed new challenges to the global cardiovascular community. The goals of any tertiary cardiac center are 2-fold: limit transmission of the infection to the public and healthcare personnel while providing timely and safe care to patients with acute coronary syndromes (ACS). Conventionally, the role of cardiovascular computed tomography (CCT) in ACS has been minimal as the majority of these patients commonly undergo an early invasive strategy. COVID19 created a new reality in many healthcare systems where an invasive strategy is limited by the availability of healthcare workers, personal protective equipment (PPE) and beds. In this study we review the evolving role of CCT in ACS in a tertiary cardiac center in Saudi Arabia during the COVID19 pandemic.

## Methods

This is a single center snapshot study of the first 27 consecutive patients who presented with chest pain or ACS and underwent CCT evaluation at the Cardiac Center of King Fahd Armed Forces Hospital (KFAFH) after the lockdown in the city of Jeddah (March 23, 2020). Research and ethics committee approval was obtained prior to the data collection. Total CCT studies, patient disposition and healthcare personnels' infection rates were prospectively collected from the center's key performance indicator database, infection control database and employee health records. All baseline characteristics, assigned strategy, outcome and COVID19 status were obtained from the patients' electronic medical record.

**Figure 1:**
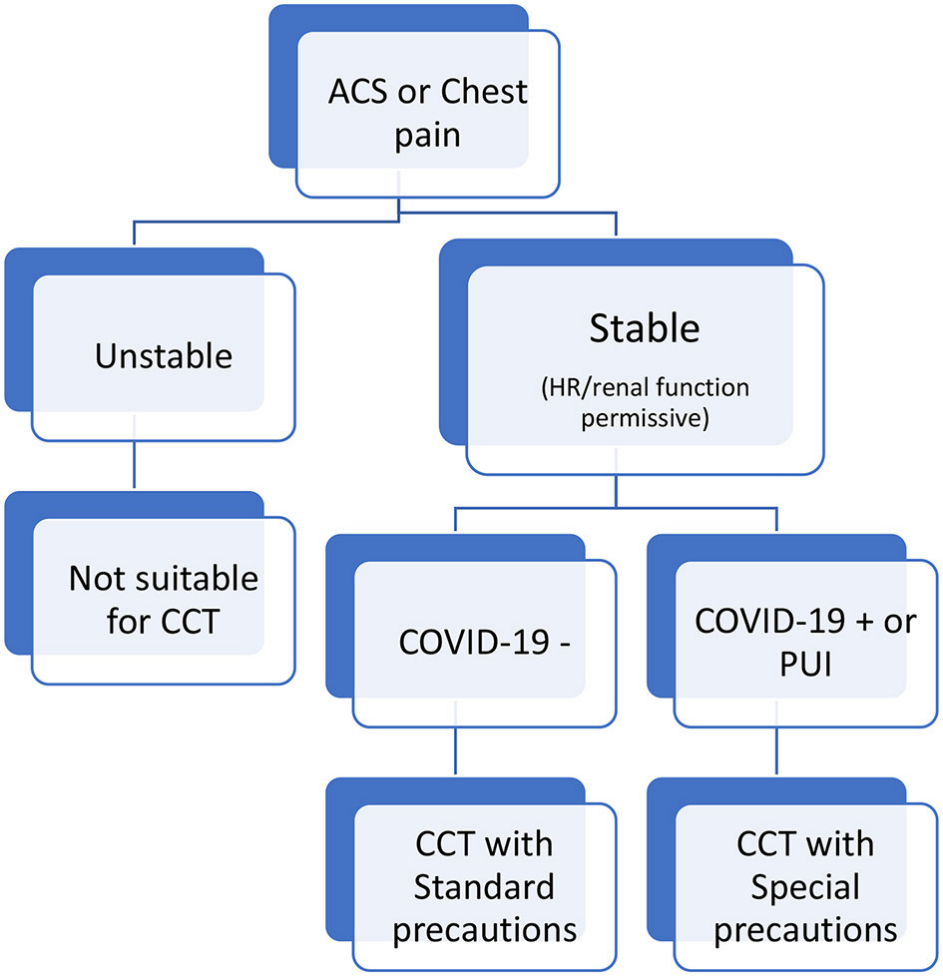
Chest pain or acute coronary syndrome in the emergency room. *All ST Elevation Myocardial Infraction (STEMI) patients were excluded.

All elective admissions to the cardiac center were canceled. Only patients requiring acute cardiac care were transferred from outlying hospitals provided the COVID19 screen was negative. Patients presenting to KFAFH's emergency room with an ACS were screened for COVID19 symptoms in particular fever, cough and shortness of breath. If there was no suspicion of COVID19, the usual guideline directed ACS protocols were adopted. However, those with a suspicion based on symptoms or referral source were placed in isolation and a screening test was requested. The COVID19 polymerase chain reaction (PCR) was sent to an on-site laboratory and results were obtained in 3–4 h. The ACS CCT protocol adopted is defined in Figure 1. Those with chest pain or an ACS were risk stratified according to the GRACE Score and hemodynamics. If patients were unstable, had an ST elevation myocardial infarction (STEMI) or had prohibitive renal dysfunction, they were excluded and did not undergo a CCT. Stable patients with chest pain or an ACS underwent CCT. The precautions employed for those with a COVID19 negative screen were standard universal precautions. Those under investigation or positive for COVID19 underwent CTA in a dedicated Seimens 128 Flash CT Scanner with special precautions. During the CCT study, staffing in the room was minimized to the nurse who placed the electrodes, connected the intravenous cannula and positioned the patient. The nurse wore PPE as recommended by the WHO including an N95 mask. The imaging expert and radiographer remained in the control room. The patient was transported with a regular surgical mask directly to the one designated CT scanner bypassing the holding and recovery areas.

**Figure 2:**
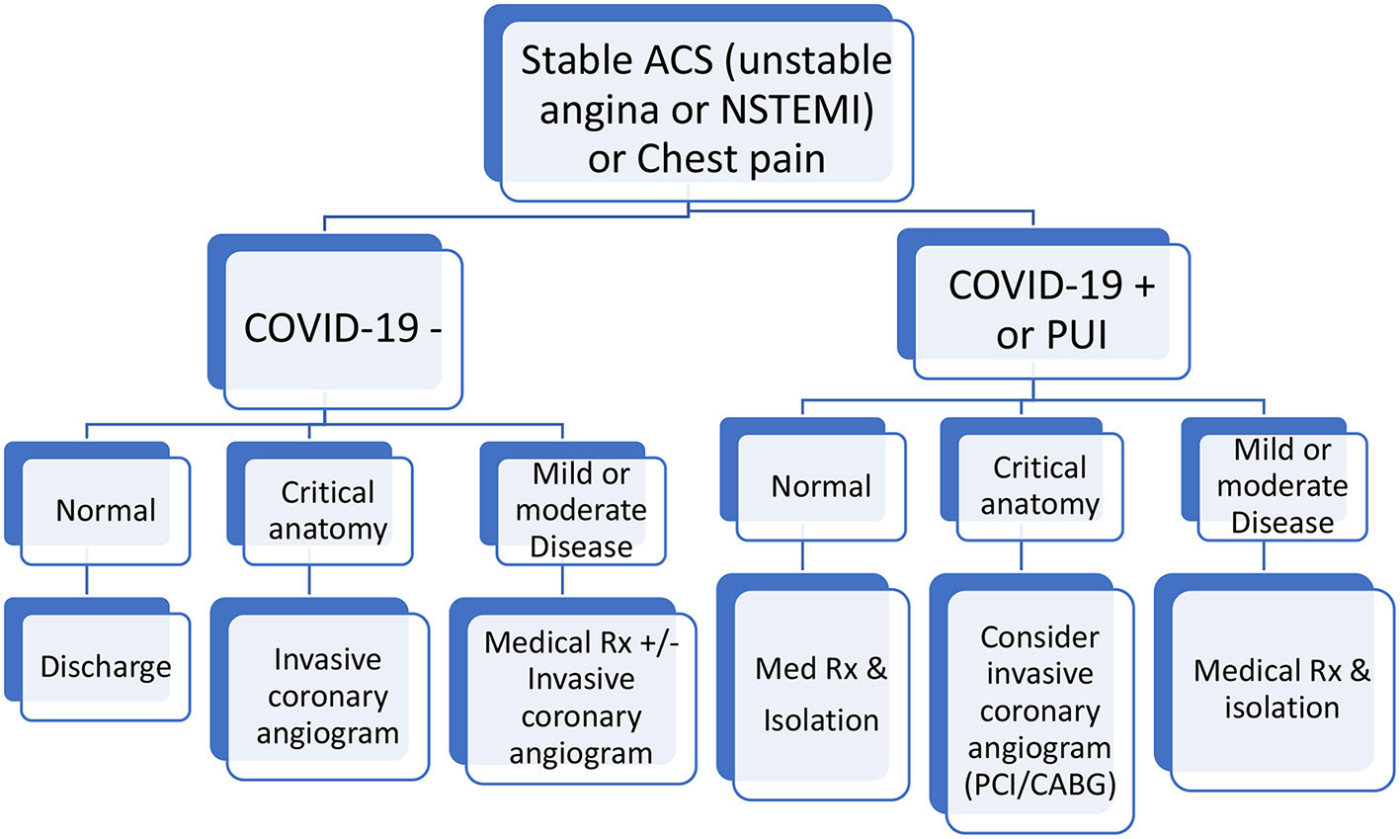
Chest pain or acute coronary syndrome clinical pathway. *All ST elevation myocardial infraction (STEMI) and unstable patients were excluded.

Patients' COVID19 status and the results of the CCT were then used to determine the treatment strategy as illustrated in Figure 2. Patients who had a negative COVID19 PCR and a normal CCT were discharged home immediately. Those with critical anatomy were admitted for an invasive coronary angiogram. Critical anatomy was defined as stenosis >70% in major epicardial coronary vessels or Left main stenosis. Those with mild or moderate distal or branch vessel disease were discussed with the main responsible physician and patient to consider medical therapy and early discharge. As for those who were COVID19 positive or under investigation, normal or mild to moderate coronary disease on CCT allowed discharge of the patients to an isolation facility. Critical anatomy required hospitalization in an isolation unit. A case by case discussion ensued in a heart team format to determine the most appropriate course of action (conservative, surgical or percutaneous revascularization).

The CCT study was performed using standard imaging protocols for coronary evaluation and included the lung fields. Two-dimensional maximum intensity projections and multiplanar reformatted images as well as three-dimensional images were evaluated on a Syngo Via workstation by an imaging expert. Coronary artery calcium score, coronary stenosis and the lung parenchyma were all assessed and reported. Quadruple rule outs for pulmonary embolism, myocardial perfusion, coronary anatomy and parenchymal lung involvement were employed whenever possible.

### Statistical Analysis

In this prospective analysis the continuous variables are presented as mean and range. The qualitative variables are presented in percentages.

## Results

This cohort comprises the first 27 patients evaluated in the emergency room immediately after the COVID19 lockdown in Jeddah, March 15, 2020 who presented with acute onset chest pain consistent with angina of which 13 had a positive high sensitivity troponin I assay. The results are summarized in Table 1. The mean age was 52 years (range 20–73 years). Fifty-two percent were women. Six patients had no known atherosclerotic cardiovascular risk factors and seven had 3 or more risk factors. The mean left ventricular ejection fraction was 50% (range 15–65%). All CCT images were interpretable with no limitations or significant artifact. CCT identified critical disease in 7 patients (26%) all of whom had presented with a NSTEMI, normal epicardial coronary arteries in 11 (41%) and mild to moderate disease in 9 (33%). All patients with normal or mild to moderate disease were assigned to a conservative strategy and discharged within 24 h. Those with a NSTEMI and critical anatomy were assigned to an additional invasive evaluation of which four underwent ad *hoc* percutaneous coronary interventions (PCI) for the Left anterior descending artery (3) and Left Circumflex artery (1), 1 had in-hospital coronary artery bypass grafting (CABG) for distal Left Main disease and two were deferred for elective CABG at a later date for three vessel disease. Examples of obstructive disease detected by CCT and treated by PCI are illustrated in Supplementary Figures 1, 2.

**Table 1:** Summary of results.

**General characteristics**	**Total 27**
Age (years)	Range 20–73 (Mean 52)
Gender – Female	14 (52 %)
**ASCVD risk factors**	
0	6
1	6
2	8
>3	7
**Presentation of ACS**	
Chest pain/Unstable Angina	14
NSTEMI	13
**CT findings**	
Normal	11
Mild to Moderate	9
Critical	7
**Treatment strategy**	
PCI (In-patient)	4
CABG (In-patient)	1
CABG (Deferred)	2
Conservative	20
EF (mean)	Range 15–65 % (Mean 50%)
Length of Hospital Stay	0–5 days (Mean 1 day)
Chest pain/Unstable Angina	0–2 days (Mean 0 days)
NSTEMI	0–5 days (Mean 1.6 days)

## Discussion

CCT has an established role in the assessment of patients with stable coronary syndromes which has been validated in multiple randomized clinical trials (1–3). It's role in the evaluation of patients in the emergency room has also been studied in several randomized trials the most notable being the ROMICAT I and II trials (4–7). However, the patients in these trials were low to intermediate risk with negative initial cardiac serum markers and electrocardiograms. In the ROMICAT cohorts, both the feasibility and safety of such a strategy were confirmed. The average length of stay was significantly reduced with no increase in major adverse cardiac events at 28 days.

During the current COVID19 pandemic, utilization of healthcare resources including beds and PPE in addition to exposure of HCW to COVID19 is a priority (8, 9). This must be balanced against a timely designation of the treatment strategy for ACS patients. We, therefore, included those with positive serum markers who had either confirmed or suspected COVID19 infection. The results of this snapshot study indicated that 80% of patients were discharged within 24 h, the majority directly from the emergency room signifying very efficient bed utilization. Fifty perchantage of those were discharged the same day and carried a low Grace Score. The overall low Grace Score of the study population, including those who presented with a NSTEMI, suggests such a strategy of non-invasive assessment of the coronary arteries and rapid discharge planning may be applicable only to a low or intermediate risk populations. This is in line with the previously mentioned ROMICAT studies enrolling similar populations and excluded high risk, STEMI and patients in shock. There was a marked reduction in invasive angiography and subsequently less consumption of PPE and exposure of catheterization personnel. None of the staff in the CCT department or catheterization laboratory were infected.

Furthermore, troponin elevation in critically ill patients is frequently due to Type II myocardial infarction unrelated to obstructive epicardial coronary artery disease. This protocol was able to identify those with critical anatomy that required invasive angiography and revascularization. Those who didn't have critical anatomy, the CCT was able to furnish additional information such as evaluation of the lung fields for COVID19 pneumonitis, myocarditis and pulmonary embolism all which could raise troponins and have been reported in COVID19 cases.

There is ample evidence that the negative predictive value (NPV) of CCT exceeds 90% sensitivity and specificity of 96.5 and 72.4% respectively. It is noteworthy that the NPV is not impacted by the clinical risk scores (10). The utility in low risk ACS has been added to the European Society of Cardiology's Non-ST elevation Myocardial Infarction Guidelines (ESC NSTEACS) elaborated in August 2020 suggesting a wider role for this technology in select patients. Our experience during the COVID19 adds to the growing experience with streamlining care using CCT in low risk ACS (11). It should be noted that even the ESC guidelines recognize that non-obstructive coronary disease carries prognostic implications. Intracoronary imaging and cardiac magnetic resonance imaging have been recommended in the evaluation of those with myocardial infarction and non-obstructive coronary arteries (MINOCA). Establishing protocols that permit expeditious inpatient or outpatient assessment of such cases is important.

### Limitations

This is a snapshot study with a small number of patients. The study only serves as a pilot that requires further validation through large scale randomized trials. There is no long term follow up available. However, during such an outbreak the goal of all centers is to provide acute care for a surge of COVID19 patients that can overwhelm the system. Furthermore, it should be noted that the ROMICAT trials reported outcome data at 28 days only. The purpose of pathways that include CCT is to enable cardiac centers to further risk stratify patients and allow early discharge. CT scanners are often shared by the cardiology and radiology services. Therefore, adoption of these pathways would require coordination between the departments. In addition, during such an outbreak it is necessary to prioritize studies for all patients with both cardiac and non-cardiac needs.

## Conclusions

COVID19 presented a unique opportunity for CCT to assist in providing anatomic risk stratification for those with ACS allowing expedited discharge of those with low risk anatomy and preserving beds during the outbreak. It also reduced invasive procedures and exposure of catheterization laboratory personnel with conservation of PPE in lower risk patients. Nevertheless, individualized case by case decisions are still necessary to ensure patient outcomes for the higher risk categories. This snapshot warrants exploring the role of CCT in ACS further through larger randomized studies. It is in keeping with the latest ESC NSTEACS guidelines.

## Data Availability Statement

The raw data supporting the conclusions of this article will be made available by the authors, without undue reservation.

## Ethics Statement

The studies involving human participants were reviewed and approved by Ethics Committee of King Fahd Armed Forces Hospital. Written informed consent for participation was not required for this study in accordance with the national legislation and the institutional requirements.

## Author Contributions

All authors listed have made a substantial, direct and intellectual contribution to the work, and approved it for publication.

## Conflict of Interest

The authors declare that the research was conducted in the absence of any commercial or financial relationships that could be construed as a potential conflict of interest.
