# Supplementary figures and images for: Role of Cardiovascular Computed Tomography in Acute Coronary Syndromes During the COVID-19 Pandemic-Single Center Snapshot Study
Source: Front Cardiovasc Med. 2021 May 11;8:665735. doi: 10.3389/fcvm.2021.665735 (PMC8144287; doi:10.3389/fcvm.2021.665735)

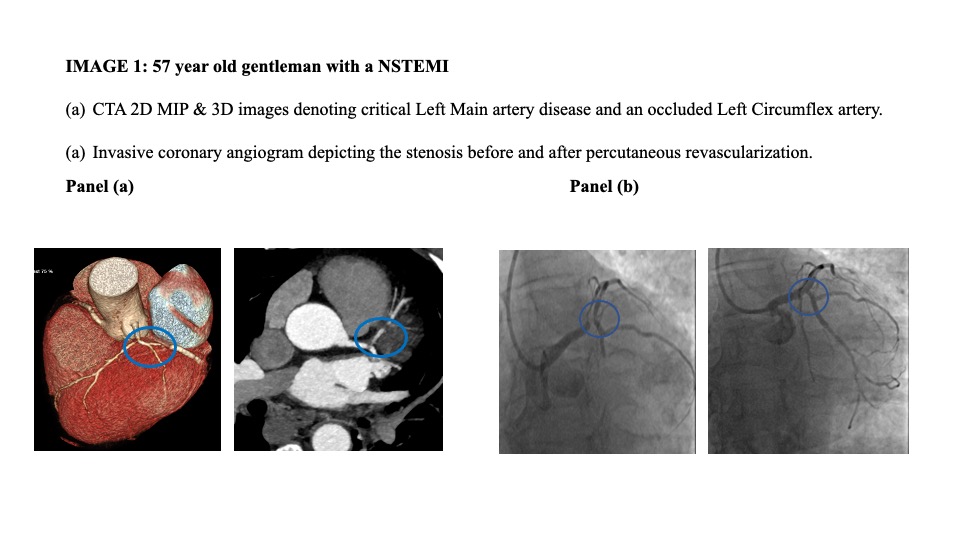

Supplement: Supplementary file 1 [file Image_1.JPEG]

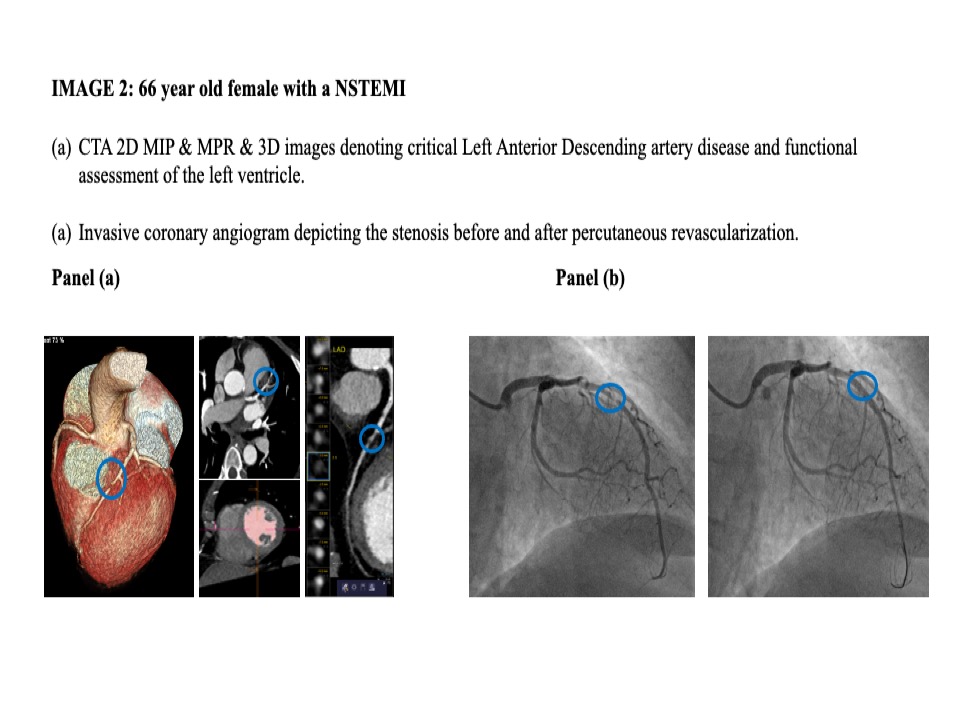

Supplement: Supplementary file 2 [file Image_2.JPEG]
